# Contact-Angle Hysteresis and Contact-Line Friction on Slippery Liquid-like Surfaces

**DOI:** 10.1021/acs.langmuir.0c02668

**Published:** 2020-12-01

**Authors:** Hernán Barrio-Zhang, Élfego Ruiz-Gutiérrez, Steven Armstrong, Glen McHale, Gary G. Wells, Rodrigo Ledesma-Aguilar

**Affiliations:** † Smart Materials and Surfaces Laboratory, Northumbria University, Newcastle upon Tyne NE1 8ST, United Kingdom; ‡ Institute for Multiscale Thermofluids, School of Engineering, University of Edinburgh, The King’s Buildings, Mayfield Road, Edinburgh EH9 3FB, United Kingdom

## Abstract

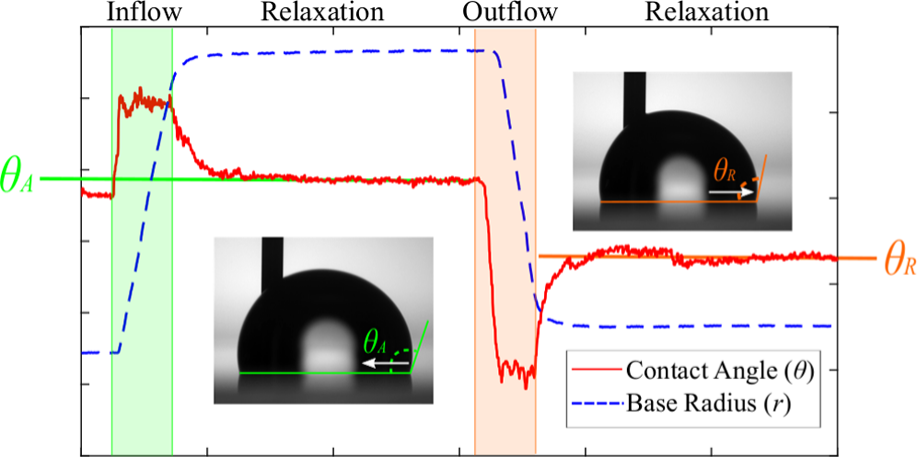

Contact-line pinning and dynamic friction are fundamental forces that oppose the motion of droplets on solid surfaces. Everyday experience suggests that if a solid surface offers low contact-line pinning, it will also impart a relatively low dynamic friction to a moving droplet. Examples of such surfaces are superhydrophobic, slippery porous liquid-infused, and lubricant-impregnated surfaces. Here, however, we show that slippery omniphobic covalently attached liquid-like (SOCAL) surfaces have a remarkable combination of contact-angle hysteresis and contact-line friction properties, which lead to very low droplet pinning but high dynamic friction against the motion of droplets. We present experiments of the response of water droplets to changes in volume at controlled temperature and humidity conditions, which we separately compare to the predictions of a hydrodynamic model and a contact-line model based on molecular kinetic theory. Our results show that SOCAL surfaces offer very low contact-angle hysteresis, between 1 and 3°, but an unexpectedly high dynamic friction controlled by the contact line, where the typical relaxation time scale is on the order of seconds, 4 orders of magnitude larger than the prediction of the classical hydrodynamic model. Our results highlight the remarkable wettability of SOCAL surfaces and their potential application as low-pinning, slow droplet shedding surfaces.

## Introduction

The interaction of droplets with engineered solid surfaces has relevance from both a fundamental and an applied perspective. On the one hand, understanding the mechanisms involved in the interaction between droplets and complex surfaces can unveil new physics in the context of solid–liquid interactions. On the other hand, engineered surfaces can be used to solve problems in applications such as ink-jet printing,^1^ coating,^2^ and lubrication.^3^

Recently, there has been a sustained interest in slippery omniphobic covalently attached liquid-like (SOCAL) surfaces, which are a type of engineered, ultrasmooth solid surface that offers remarkably low static friction to the motion of droplets.^4−6^ SOCAL surfaces are achieved by acid-catalyzed graft polycondensation of dimethyldimethoxysilane, where short polymer chains are covalently bound to a solid substrate creating a nanometric monolayer that shields a droplet from the underlying solid substrate.^4^ The polymer coating of a SOCAL surface plays a similar role to the intermediary liquid lubricant film used to create slippery liquid-infused porous surfaces (SLIPS)^7^ and lubricant-impregnated surfaces (LIS):^8^ it creates a smooth surface that masks the chemical and topographical heterogeneity of the solid substrate. However, unlike SLIPS or LIS, on SOCAL surfaces, a droplet is in contact with a polymer coating covalently attached to the solid and not with a liquid layer. On SOCAL surfaces, droplets are subject to a very low contact-angle hysteresis, typically of 1° or below. Despite this low hysteresis, droplets on SOCAL surfaces exhibit a remarkably low mobility,^5^ indicating an unexpected high dynamic friction imparted by the surface on a moving droplet. From a fundamental perspective, this raises important questions about the physical mechanism governing the motion of contact lines on SOCAL surfaces. On the other hand, the remarkable combination of low static friction but high dynamic friction can unlock applications in surface engineering, where SOCAL surfaces act as “low-pinning-slow shedding” coatings.

In this paper, we study the static and dynamic friction of water droplets on SOCAL surfaces. We start by reviewing relevant concepts in the study of statics and dynamics of sessile droplets on solid surfaces. We report experiments of the droplet transition to a steady state driven by either an inflow or an outflow at a fixed flow rate and the subsequent relaxation to equilibrium once the flow is suppressed. We characterize static friction using the relaxation of the contact line toward a static configuration, which allows us to measure the contact-angle hysteresis directly from measurements of the apparent contact angle. In the limit of mechanical and thermodynamic equilibria, corresponding to a vanishing contact-line velocity and high relative humidity (94%), we measure well-defined, reproducible values of the advancing and receding contact angles, which yield a contact-angle hysteresis as low as Δθ = 2.1 ± 0.4°. Out of thermodynamic equilibrium, we show that the apparent contact angle deviates from the advancing and receding values due to the effect of evaporation. Out of mechanical equilibrium but at high relative humidity, we find a variation of the apparent contact angle with interface velocity. The corresponding relaxation time to mechanical equilibrium is in good agreement with an analytical model based on molecular kinetic theory.^9^

## Statics and Dynamics of Droplets on Solid Surfaces

### Statics

Consider a droplet sitting on a perfectly flat and smooth surface. Within the framework of classical thermodynamics, the equilibrium state of the droplet is given by a minimum in the total surface energy of the system. For droplets whose size is below the capillary length, this corresponds to a spherical cap shape defined by an equilibrium contact angle θ_e_, also known as Young’s angle, which is determined by the Young–Dupré equation
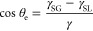
1where γ is the liquid–gas surface tension, γ_SG_ is the solid–gas surface tension, and γ_SL_ is the solid–liquid surface tension.

Equation 1 implies that the equilibrium contact angle is uniquely determined by the combination of the surface tensions. However, this assertion is only valid in the ideal case of a perfectly flat and smooth solid. In practice, any solid surface is heterogeneous at small scales because of either chemical defects or topographic roughness. Therefore, instead of a unique equilibrium contact angle, one observes a static contact angle, θ_S_, which varies over a range controlled by the surface heterogeneity.

An important consequence of the heterogeneity of a solid surface is contact-line pinning, which is the static friction that a droplet needs to overcome to start moving on the solid.^10^ A familiar situation where contact-line pinning is evident occurs when a droplet is placed on an incline: one observes that the droplet resists motion up to a maximum inclination angle at which point it moves down. At the onset of motion, the contact angle of an advancing liquid–gas interface is referred to as the advancing contact angle, θ_A_. Similarly, the contact angle at the onset of a receding motion is called the receding angle, θ_R_. Therefore, the range of the static contact angle, θ_S_, is given by
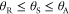
2and the amplitude of this range is a measure of the hysteresis caused by the surface heterogeneity, typically called the contact-angle hysteresis
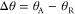
3The importance of contact-angle hysteresis becomes evident when considering the pinning force acting on a droplet. At the onset of motion, the net force acting on the contact line is given by
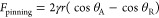
4where *r* is the base radius of the droplet.^5^ From eqs 2 and 3, it follows that the advancing and receding angles obey θ_A_ = θ_S_ + *f*Δθ and θ_R_ = θ_S_ – (1 – *f*) Δθ, where 0 ≤ *f* ≤ 1. Inserting these expressions in eq 4 and expanding in powers of Δθ gives
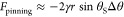
5Hence, the pinning force scales with contact-angle hysteresis by a factor determined by the normal component of the surface tension force, γ sin θ_S_.

### Relaxation to Equilibrium

Beyond the onset of motion, the shape of the droplet can be characterized in terms of a dynamic angle, θ(*v*), which depends on the velocity of the contact line, *v*.^10,11^ For an advancing contact line, the dynamic angle is higher than the advancing angle, i.e., θ(*v*) > θ_A_, and one expects that θ approaches θ_A_ as *v* → 0. Similarly, for a receding contact line, θ(*v*) < θ_R_, and θ → θ_R_ as the contact line comes to a rest.

The deviation of the dynamic contact angle from the static value is governed by the competition between driving and dissipative forces. On the one hand, the large-scale deformation of the liquid–gas interface is governed by the competition between viscous stresses and surface tension. This is described by the Cox–Voinov theory,^12,13^ which gives the following prediction of the apparent contact angle as a function of the velocity of the interface
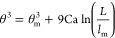
6where Ca = η*v*/γ is the capillary number, *L* is the typical macroscopic length scale where the dynamic contact angle is measured, and θ_m_ is the microscopic contact angle, measured at a microscopic cutoff length scale *l*_m_.^11^

In addition, the effect of the solid surface on the motion of the contact line is controlled by microscopic processes. Haynes and Blake developed a model for the contact-line dynamics based on molecular kinetic theory (MKT),^14^ which was subsequently used to describe the spreading of droplets on solid surfaces.^15^ In the framework of MKT, the contact-line motion is governed by the rate of adsorption and desorption of molecules from the solid. The balance between both processes sets the contact-line velocity^5^
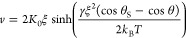
7where *K*_0_ is the frequency of adsorption–desorption of molecules at the contact line, ξ is the average distance of molecular displacements, and *k*_BT_ is the thermal energy.

We now study the relaxation of the droplet toward a spherical cap shape and derive separate expressions for the typical relaxation time based on the Cox–Voinov and MKT models. We start by assuming that the droplet shape is a spherical cap of instantaneous base radius *r*(*t*), contact-line velocity *v* = *ṙ*, and spatially uniform dynamic contact angle θ(*t*). Therefore, deviations of the droplet shape from the static configuration can be quantified in terms of the deformation angle
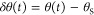
8where θ_S_ is the limiting static value of the contact angle, i.e., either θ_A_ or θ_R_ depending on whether the contact line is advancing or receding during the relaxation process. In the limit of small deformations, we expect that the velocity of the contact line varies linearly with δθ, i.e.
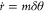
9where the constant *m* is determined by the physical mechanism governing the motion of the contact line. For a spherical cap, one has the geometrical relation
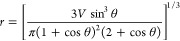
10Expanding this expression in powers of δθ and differentiating with respect to time lead to the relation
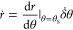
11Combining eqs 9 and 11 and integrating with respect to time give the exponential relaxation
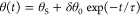
12where δθ_0_ is the initial deformation and
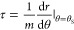
13is the relaxation time.

For viscous-dominated dynamics, the microscopic contact angle is expected to be close to the static value,^11^ i.e., θ_m_ ≈ θ_S_. Setting θ = θ_S_ + δθ in eq 6, expanding in powers of δθ, and using eqs 9 and 13 lead to the following expressions
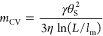
14and
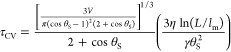
15One can obtain equivalent expressions using the MKT model. Expanding eq 7 in powers of δθ and using eqs 9 and 13, we obtain
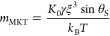
16and
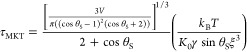
17

## Experimental Methods

### SOCAL Surfaces

SOCAL surfaces were prepared following the methodology outlined by Wang and McCarthy^4^ and optimized using the experimental parameters reported by Armstrong et al.^6^ Glass slides (25 mm × 75 mm) were cleaned in a solution of deionized (DI) water and detergent (Decon 90, 2% solution) placed into a 30 min ultrasonic bath followed by rinsing with DI water, acetone, and isopropanol (IPA). The clean slides were then put in an air plasma oven (Henniker HPT-100) operating at a power of 30 W for 30 min, which creates OH^–^ radicals on the glass substrate. The slides were immersed for 5 s in a solution of isopropanol, dimethyldimethoxysilane, and sulfuric acid (100, 10, and 1 wt %, respectively) and withdrawn manually. This solution reacts with the exposed OH^–^ groups, inducing the polycondensation of PDMS chains on the surface. The result is the grafting of an ∼4 nm thick liquid-like polymer coating on the surface of the glass substrate.^4^

### Contact-Angle Measurements

**Figure 1:**
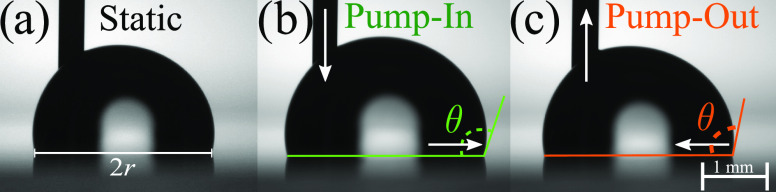
Experimental setup. (a) Droplet of controlled initial volume *V* is placed on a SOCAL surface and connected to a micropump through a thin needle. (b, c) Micropump injects or withdraws liquid at a prescribed flow rate *q̇* (vertical arrows). The instantaneous apparent contact angle, θ, and base radius, *r*, are measured using image analysis. The scale bar is 1 mm.

Figure 1a shows the experimental setup. A SOCAL surface sample is positioned within a drop shape analyzer (Krüss, DSA25), equipped with a leveling stage, a thermostat, and humidity control. The experimental procedure consists of depositing a droplet of deionized water and controlled volume, *V* = 8 μL, on the SOCAL surface. A thin needle (outer diameter: 0.4 mm) is connected to a micropump (Cellix ExiGo) and used to feed or withdraw liquid from the edge of the droplet. At the same time, the apparent contact angle is measured at the opposite edge of the drop, where the droplet maintains a shape close to a spherical cap. The volume variation is carried out as follows. A volume Δ*V* = 4 μL of water is first injected into the droplet at a prescribed flow rate, *q̇*, which we vary between 1 and 10 μL/min (Figure 1b). The droplet is then left to rest with the needle in for a period of 2 min to allow the contact line enough time to return to a static position. Subsequently, a volume Δ*V* = 4 μL of water is withdrawn from the droplet at the same flow rate (Figure 1c) and is then left to rest for 2 min before video recording is stopped. The droplet is then removed from the surface, and the process is repeated.

All experiments are performed at a controlled relative humidity, which we vary between 30 ± 0.5 and 94 ± 0.5% and at a constant temperature, *T* = 25 ± 0.2 °C. For each set of parameters, the experiment is repeated 5 times.

The experiments were recorded using a video camera, and the resulting images were analyzed using pyDSA, an in-house droplet shape analyzer.^16^ The resolution of the video footage was at least 2 pixels/μm, and the apparent contact angle of the droplet is determined by image analysis as follows. First, the apparent contact line is detected using the droplet’s reflection on the solid. The droplet’s free contour is determined using a brightness threshold function. A third-degree polynomial is fitted to the contour of the droplet over a region that ranges from the free edge of the drop to the point where the needle meets the droplet. The algorithm then determines the point at which the polynomial meets the contact line and computes the apparent contact angle as the local slope. The resolution of the images allows the algorithm to produce droplet contours formed by ∼250–500 points, leading to a small fitting error. Therefore, the systematic measurement error in the apparent angle is δθ ∼ 0.2°, which is commensurate with previous errors reported in the literature.^17,18^

To determine the advancing and receding contact angles and, therefore, the contact-angle hysteresis, we used two different methods. As a first method, we determined the onset of motion of the contact line upon increasing and decreasing the volume of the droplet.^10,19−22^ This point is then mapped to the corresponding apparent contact angle: θ_A_ for a volume increase and θ_R_ for a volume decrease. The second method consists of tracking the apparent contact angle as the velocity of the contact line vanishes after a change in volume, and identifying the corresponding limiting value of the apparent contact angle as either the advancing or the receding angle.^23^

## Experimental Results

**Figure 2:**
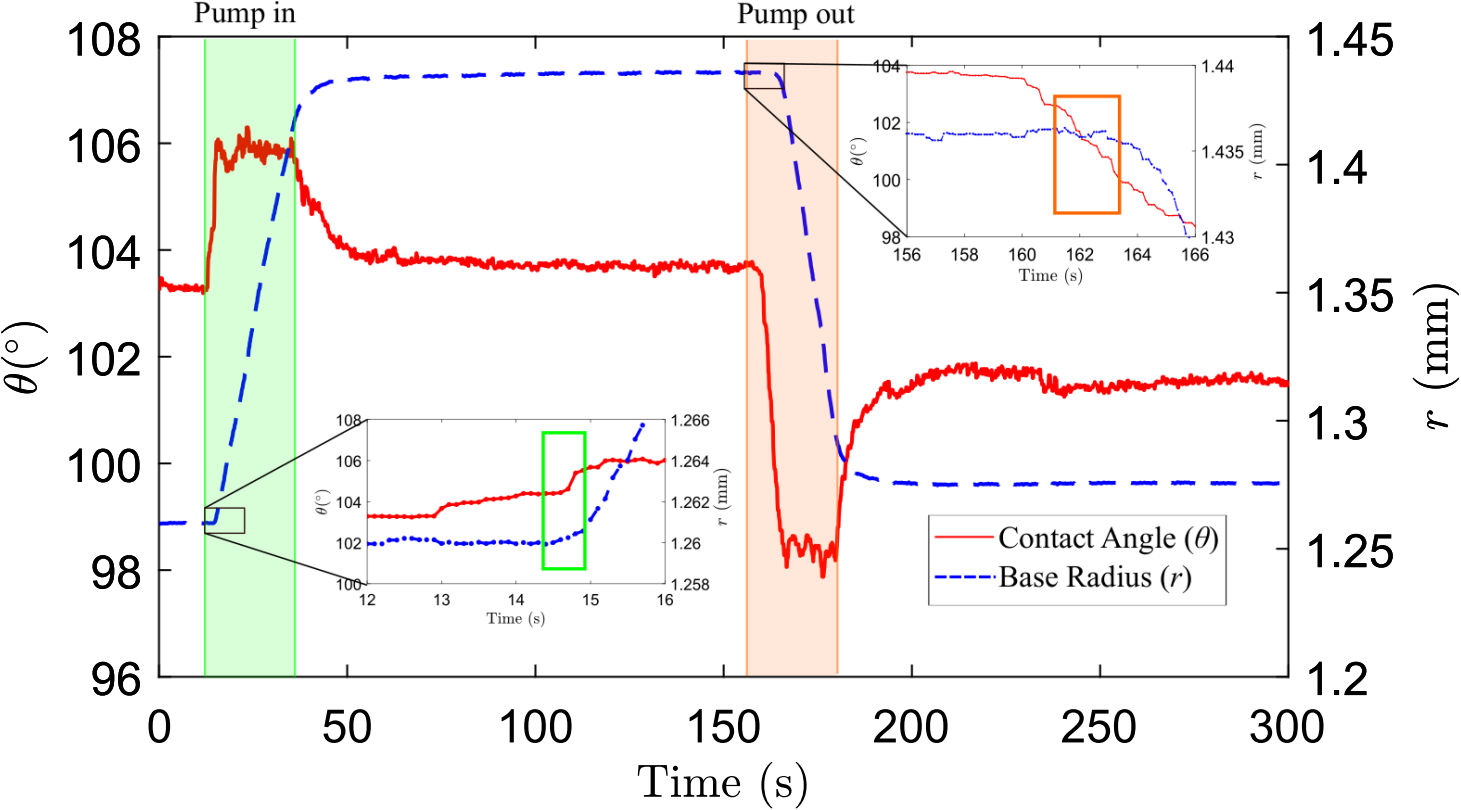
Apparent contact angle and base radius measurements at high relative humidity. The graph of a typical experimental set of data performed at a constant flow rate *q̇* = 10 μL/min at *T* = 25° C and RH = 94%. The zoomed-in regions show how the smooth transition from a static to a moving contact line introduces uncertainty in the measurement of the advancing and receding angles.

Figure 2 shows representative measurements of θ(*t*) (red line) and *r*(*t*) (blue line) for an 8 μL droplet subject to changes in volume at a constant flow rate (Δ*V* = ±4 μL; *q̇* = 10 μL/min), followed by relaxation periods at a zero flow rate (Δ*t* = 120 s). The temperature and relative humidity are fixed at *T* = 25 °C and RH = 94%, ensuring that the droplet does not undergo significant evaporation during the experiment. During the injection phase (green-shaded region), the apparent contact angle increases sharply from the initial value θ_i_ ≈ 103°. This sharp increase is followed by a steady motion of the contact line, where θ ≈ 106° and where the base radius grows at a rate *ṙ* = 9 ± 1 μm/s. A similar situation occurs during the withdrawal phase of the experiment (red-shaded region), where the apparent contact angle sharply falls as the contact line starts to recede until it settles at θ ≈ 99° for a contact-line velocity, *ṙ* = 12 ± 1 μm/s. Once the flow is switched off, the apparent contact angle relaxes to well-defined constant values: θ = 103.8 ° after injection and θ = 101.6° after withdrawal.

### Effect of Flow Rate

The relaxation of the apparent contact angle reported in Figure 2 indicates that dynamical effects due to a finite flow rate affect the shape of the droplet.^23^ To understand the relevance of this effect for droplets on SOCAL surfaces, we performed experiments on a fresh SOCAL sample considering three different flow rates: *q̇* = 1, 5, and 10 μL/min. As before, the experiment consisted of a change in the droplet volume Δ*V* = ±4 μL, followed by a relaxation at a zero flow rate (Δ*t* = 300 s). The experiment was repeated three times for each flow rate. The temperature and relative humidity were kept at *T* = 25 °C and RH = 94%.

**Figure 3:**
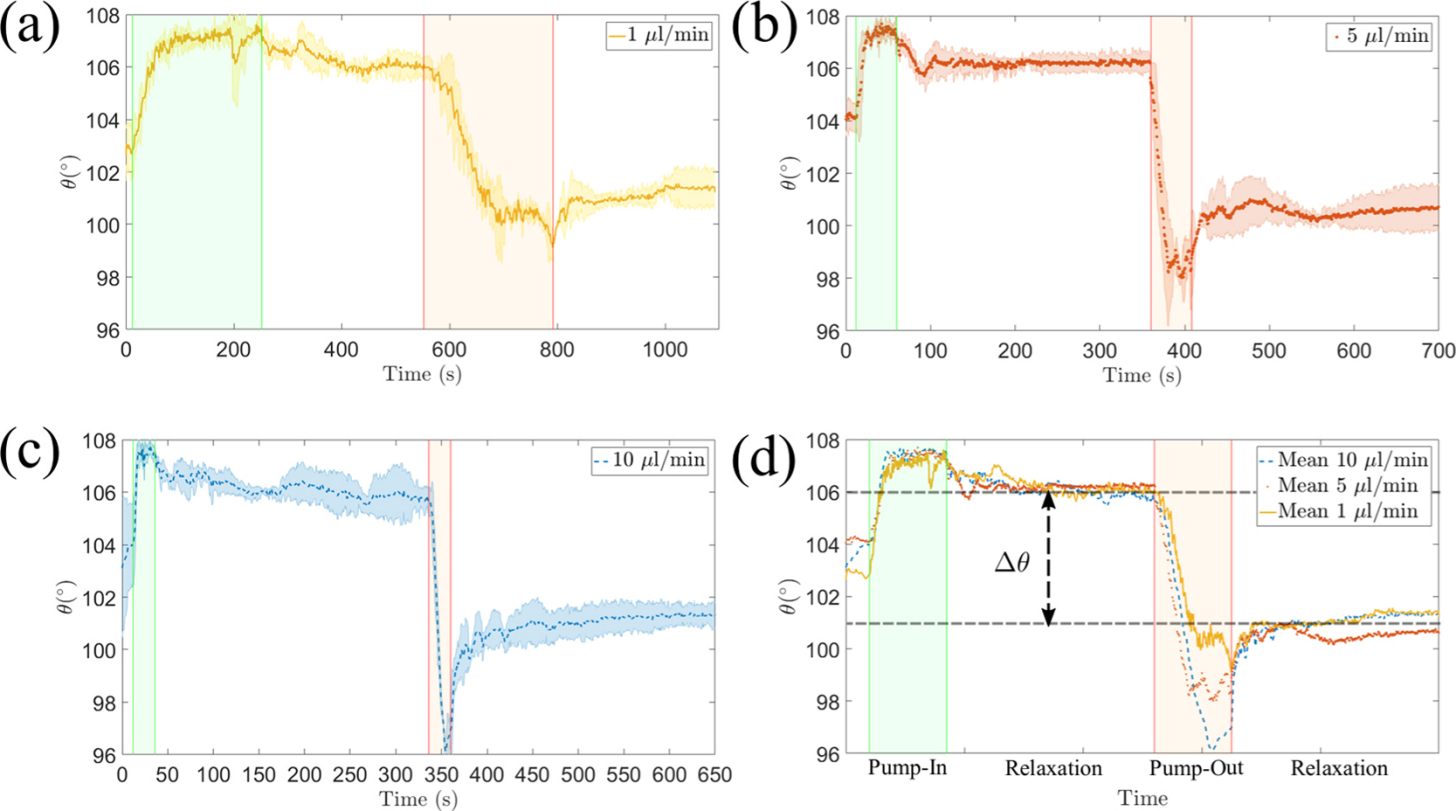
Effect of flow rate on the apparent contact angle. (a–c) Variation of the contact angle at different flow rates. (d) Overlap of the experimental data. The apparent contact angle relaxes to constant values, which are independent of the flow rate. The difference between these values is identified as the contact-angle hysteresis.

Figure 3a–c shows measurements of the apparent contact angle. As before, we observe two dynamical regimes, corresponding to an increase or a decrease in the base radius, which are characterized by maximum and minimum values of the apparent contact angle, respectively. These regimes are followed by a relaxation to static values. Figure 3d shows a superposition of the data for the three flow rates studied. In the plot, we use arbitrary units of time to match the volume increase/decrease windows while we leave the rest of the time data unaltered (i.e., time units in the relaxation portions of the plot are the same for all flow rates). Although the effect of the input and output rates is subtle, it is clear that, in all cases, the response of the apparent contact angle during a change in the droplet volume depends on the flow rate. In contrast, the relaxation at zero flow rate consistently leads to the same relaxation curves and limiting static values of the apparent contact angle regardless of the flow rate.

### Effect of Relative Humidity

**Figure 4:**
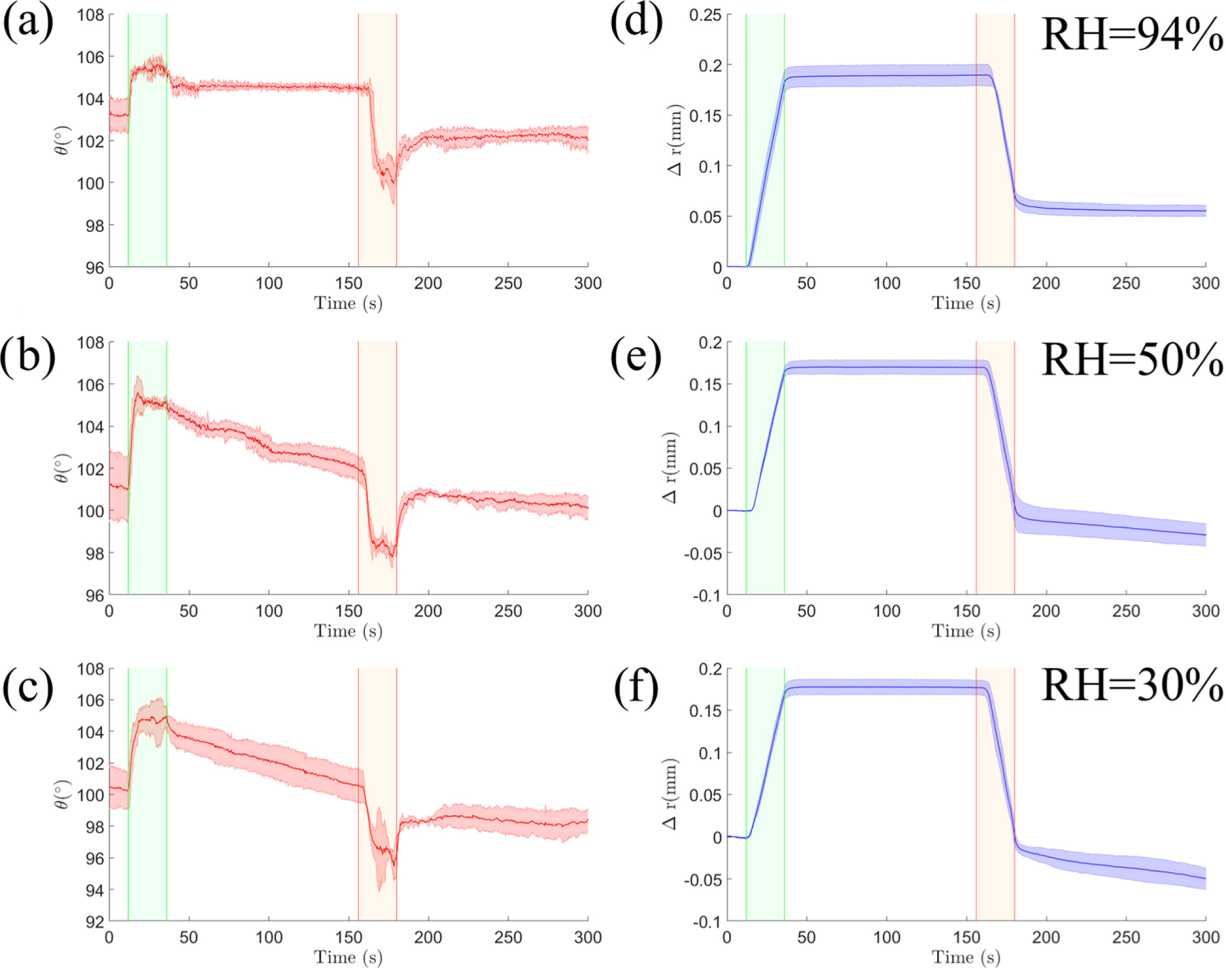
Influence of relative humidity on the apparent contact angle and the base radius. (a–c) Variation of the apparent contact angle at RH = 94, 50, and 30%, respectively. (d–f) Corresponding change in the droplet base radius.

To understand the effect of relative humidity on the droplet’s apparent contact angle, we carried out experiments at RH = 94, 50, and 30%, at a fixed flow rate, *q̇* = 10 μL/min, and temperature, *T* = 25 °C. For each experiment, the relaxation window was kept at Δ*t* = 120 s. Figure 4 shows the changes in the apparent contact angle (a–c) and base radius (d–f) for the three relative humidities considered. We report the change in base radius, Δ*r* = *r* – *r*_0_, to account for variations in the initial radius, *r*_0_. During the injection phase, the apparent contact angle reaches the same dynamic value regardless of the relative humidity θ = 105 ± 1.1°. However, during the subsequent relaxation, there is a significant change in the apparent contact angle at different relative humidities. Unlike the plateau behavior observed at RH = 94%, at RH = 50 and 30%, the apparent contact angle decreases with time at a rate that becomes stronger with decreasing relative humidity. During the same step, the base radius remains constant and independent of the relative humidity (see panels d–f in Figure 4). In the withdrawal phase, we observe an initial decrease of the apparent contact angle. Once the flow is switched off, the apparent contact angle relaxes to a plateau while the base radius decreases at a roughly constant rate. Both the plateau value of the apparent contact angle and rate of change of the base radius depend on the relative humidity.

## Discussion and Analysis

### Contact-Angle Hysteresis Measurement and Uncertainty

We first discuss the uncertainty in the measurement of the advancing and receding contact angles on SOCAL surfaces and its effect on the determination of the contact-angle hysteresis.

Typically, θ_A_ and θ_R_ are identified as the apparent contact angles at the onset of motion of the contact line upon an increase or decrease of the volume of the droplet, respectively.^6,10,19−22^ On SOCAL surfaces, however, the onset motion is difficult to identify with precision. This is because, as shown in the zoomed-in regions of Figure 2, the apparent contact angle and the base radius vary smoothly as the contact line starts to move. The typical range of transition of the base radius from the static value to a constant contact-line velocity is Δ*r* ≈ 0.2 mm. The corresponding range of change in the apparent angle is Δθ ≈ 2°, which is comparable to the overall change in θ during the volume change. As shown in Table 1, the uncertainty in the measurement of the advancing and receding contact angles is on the order of 1°. This leads to a contact-angle hysteresis Δθ = 2.5 ± 1.7°.

**Table 1:** Apparent Contact-Angle Measurements of Water Droplets on SOCAL Surfaces^a^

	volume-change method			contact-line relaxation method		
trial number	θ_A_ (deg)	θ_R_ (deg)	Δθ (deg)	θ_A_ (deg)	θ_R_ (deg)	Δθ (deg)
1	104.4	100.3	4.1	103.8	101.6	2.2
2	105.5	101.3	4.2	104.2	102.2	2.0
3	104.6	104.3	0.3	104.6	102.3	2.3
4	105.4	104	1.4	104.3	102.8	1.5
5	105.1	102.4	2.7	104.9	102.3	2.6
mean (deg)	105.0	102.4	2.5	104.4	102.2	2.1
s.d. (deg)	0.5	1.7	1.7	0.4	0.4	0.4

a Volume-change method: θ_A_ and θ_R_ are determined by estimating the onset of motion of the contact line at a constant flow rate *q̇* = 10 μL/min. Contact-line relaxation method: θ_A_ and θ_R_ are determined as the limiting apparent contact angles that the droplet exhibits after relaxation to a static shape. The temperature and relative humidity are *T* = 25 °C and RH = 94%.

Shirtcliffe et al. proposed that the advancing and receding angles can only be measured in the limit of a vanishingly small flow rate.^23^ In our experiments, this limit corresponds to the relaxation of the apparent contact angle after the flow rate is stopped. Indeed, as shown in Figure 3, such a relaxation leads to the same limiting static values of the apparent contact angle regardless of the flow rate. Table 1 shows measurements of θ_A_ and θ_R_ obtained after the contact-line relaxation for the same experimental conditions of the volume-change method. The results show a significant (3-fold) reduction of the standard deviation of the measurements, which leads to a more consistent contact-angle hysteresis measurement, Δθ = 2.1 ± 0.4°.

Note that, even though the average contact-angle hysteresis obtained from both methods is similar, the relative error for the volume-change method amounts to 68%. This is clearly important, as the corresponding error in the pinning force is proportional to the error in the contact-angle hysteresis (see eq 4). In contrast, the error in the measurement of Δθ obtained from the contact-line relaxation is consistently smaller (19% for the data reported in Table 1) and confirms the low-pinning force exerted by the SOCAL surface on water droplets.

### Contact Angles In and Out of Thermodynamic Equilibrium

We now discuss the effect of relative humidity on the contact-angle hysteresis. Figure 4a,d shows measurements of the apparent contact angle and droplet base radius upon a change in volume at a high relative humidity (RH = 94%), corresponding to conditions close to thermodynamic equilibrium. After either an advancing or a receding motion of the contact line, both the apparent angle and droplet base radius relax to well-defined constant values, with no appreciable subsequent variation over the time scale of the experiments.

Figure 4b,c,e,f shows the corresponding curves for a lower relative humidity (RH = 50 and 30%). After a volume increase, the apparent contact angle undergoes a sustained decrease over time (Figure 4b,c), while the base radius of the drop remains constant (Figure 4e,f). This indicates that the droplet is out of thermodynamic equilibrium and undergoing a constant contact-area mode of evaporation.^24^ Indeed, the rate at which the apparent contact angle decreases is larger for smaller relative humidity. This is likely due to a higher mass loss due to evaporation. On the other hand, after a volume decrease, the apparent contact angle remains constant, while the base radius decreases. This is consistent with a constant contact-angle mode of evaporation.^24^ The apparent contact angle, however, is not equal to the receding contact angle measured at high relative humidity. It decreases with lower relative humidity (see Table 2). This indicates that the contact line is out of both thermodynamic and mechanical equilibria and recedes from the solid surface at a rate controlled by evaporation.

**Table 2:** Effect of Relative Humidity on the Apparent Contact Angle after a Volume Decrease

relative humidity	94%	50%	30%
θ (deg)	102.1 ± 0.3	100.5 ± 0.3	98.4 ± 0.7

### Relaxation to Equilibrium

We now compare the prediction of the Cox–Voinov theory and the molecular kinetic theory (eqs 15 and 17) to the experimental measurements of the relaxation of the droplet close to thermodynamic equilibrium (RH = 94%). As shown in Figure 2, the apparent contact angle seems to follow an exponential variation toward the limiting static value. To obtain an experimental measurement of the relaxation time, τ, we fitted the measurements of the instantaneous base radius of the droplet to the function
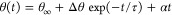
18Here, θ_∞_ corresponds to the limiting value of the contact angle after relaxation, i.e., either the advancing or receding contact angle, and Δθ is the difference between the contact angle at the initial data point of the fit with θ_∞_. The final term is introduced to account for the effect of evaporation, where α is a constant. A fit of the data to this equation yields values of α or the order of 1 × 10^–3°^/s, which leads to a variation of the contact angle of at most 0.2° over the period of relaxation. The data fits give an average relaxation time τ = 8.3 ± 5.8 s.

To obtain a prediction of the relaxation time from the Cox–Voinov theory (eq 15), we use γ = 72 mN/m, η = 0.89 mPa s, *L* = 1.2 mm, and *l*_m_ = 4 nm, where the macroscopic length scale *L* is chosen as the typical size of the droplet, and the microscopic length scale *l*_m_ is chosen to be comparable to the polymer chain length reported for SOCAL.^4,10^ This leads to τ_CV_ = 1. 131 × 10^–4^ s, which differs from the experimental measurement by several orders of magnitude. The free parameter in the Cox–Voinov model, which leads to the discrepancy, is the ratio *L*/*l*_m_ in eq 15. Keeping *L* ≈ 1 mm and fitting the Cox–Voinov theory to the experimental data give *l*_m_ ≈ 0.1 pm, which seems unrealistic.

To compare to the prediction of the molecular kinetic theory (eq 17), one needs knowledge of the frequency of the adsorption–desorption events, *K*_0_, and of the intermolecular distance, ξ. Daniel et al.^5^ studied the dissipative force exerted on water and sucrose droplets on SOCAL surfaces. By fitting their experimental data to the MKT model, they obtained *K*_0_ = 7500 s^–1^ and ξ = 3 nm. Using these values in eq 17 yields τ_MKT_ = 0. 2324 s, which is a better prediction of the experimental measurement of the relaxation time.

We now discuss the difference between the prediction from the MKT model and the experimental measurement of the relaxation time. The molecular scale, ξ, is unlikely to differ significantly from the experiments reported in ref (5). On the other hand, the experiments of ref (5) do not report a specific value of relative humidity, but it is reasonable to assume that these were carried out at ambient conditions, i.e., RH < 94%. Our experiments were carried out at a high relative humidity (RH = 94%), where the liquid is close to equilibrium with the surrounding vapor phase. Hence, we expect that the frequency of adsorption–desorption events is smaller in our experiments. Indeed, treating *K*_0_ as a single free parameter and fitting to the experimental measurement of the relaxation time yield a value *K*_0_ = 204.5 s^–1^. This suggests that at high relative humidity the contact line is slowed down by the rate of adsorption–desorption of molecules from the solid.

**Figure 5:**
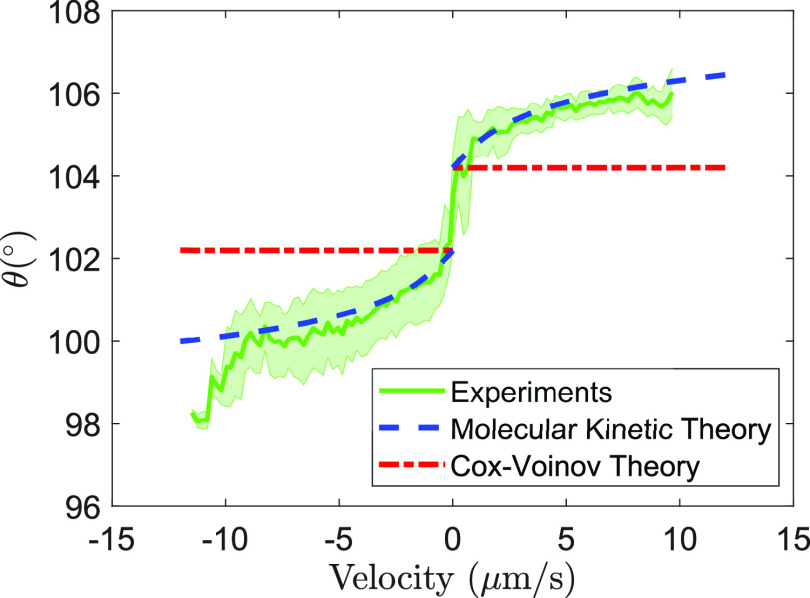
Instantaneous measurements of the contact angle vs contact-line velocity. The experimental data is averaged across five trials. The contact-angle hysteresis of the sample is Δθ = 2.1 ± 0.4°. The thick lines correspond to the predictions of the Cox–Voinov and molecular kinetic theories.

Figure 5 shows instantaneous measurements of the contact angle vs contact-line velocity averaged over five independent trials. The prediction of the Cox–Voinov theory and of the molecular kinetic theory is superimposed for comparison. For the Cox–Voinov, we use the parameter values γ = 72 mN/m, η = 0.89 mPa s, *L* = 1.2 mm, and *l*_m_ = 4 nm. For the advancing configuration, we use θ_m_ = 104.4° and for the receding configuration, we use θ_m_ = 102.2°. For MKT, we use the parameter values of *K*_0_ = 204.5 s^–1^ and ξ = 3 nm, with θ_S_ = 104.4° for the advancing configuration and θ_S_ = 102.2° for the receding configuration. The prediction of the MKT uses the parameter values fitted to match the relaxation time during the relaxation periods. The prediction of the molecular kinetic theory captures the experimental data to a better degree than that of the Cox–Voinov theory.

## Conclusions

In this work, we have studied the static and dynamic friction imparted by SOCAL surfaces on water droplets. Our study of static friction has focused on determining the contact-angle hysteresis of droplets under controlled temperature and ambient humidity conditions. We have reported direct measurements of the advancing and receding contact angles in the limit of mechanical and thermodynamic equilibria by tracking the relaxation of a droplet’s interface after a volume change. Such measurements are independent of the flow rate used to affect the volume change, leading to a significantly lower uncertainty in the measurement of the advancing and receding angles compared to the method of identifying the onset of contact-line motion.

Out of thermodynamic equilibrium, corresponding to an ambient relative humidity below the point of liquid–vapor phase coexistence, the droplet’s interface does not relax to the advancing and receding angles. Instead, the droplet undergoes evaporation keeping a constant apparent contact angle, which is always lower than the receding contact angle measured close to thermal equilibrium.

In regard to dynamic friction, we have studied the time scale of relaxation of the droplet to a static configuration and compared the experimental measurement of the relaxation time to a hydrodynamic model and a model based on the molecular kinetic theory. Our results support that the dynamic friction imparted by SOCAL surfaces on droplets is dominated not by the hydrodynamic flow close to the droplet’s edge, but by the motion of the contact line.

Our results highlight the remarkable wettability of SOCAL surfaces and can motivate further studies of the statics and dynamics of droplets on other coatings achieved by polymer grafting.^25^

## References

[ref1] de Gans B.-J.; Duineveld P. C.; Schubert U. S. Inkjet printing of polymers: State of the art and future developments. Adv. Mater. 2004, 16, 203–213. 10.1002/adma.200300385.

[ref2] Introzzi L.; Fuentes-Alventosa J. M.; Cozzolino C. A.; Trabattoni S.; Tavazzi S.; Bianchi C. L.; Schiraldi A.; Piergiovanni L.; Farris S. “Wetting enhancer” pullulan coating for antifog packaging applications. ACS Appl. Mater. Interfaces 2012, 4, 3692–3700. 10.1021/am300784n. 22758352

[ref3] Kim P.; Wong T. S.; Alvarenga J.; Kreder M. J.; Adorno-Martinez W. E.; Aizenberg J. Liquid-infused nanostructured surfaces with extreme anti-ice and anti-frost performance. ACS Nano 2012, 6, 6569–6577. 10.1021/nn302310q. 22680067

[ref4] Wang L.; McCarthy T. J. Covalently Attached Liquids: Instant Omniphobic Surfaces with Unprecedented Repellency,. Angew. Chem., Int. Ed. 2016, 55, 244–248. 10.1002/anie.201509385. 26568536

[ref5] Daniel D.; Timonen J. V.; Li R.; Velling S. J.; Kreder M. J.; Tetreault A.; Aizenberg J. Origins of Extreme Liquid Repellency on Structured, Flat, and Lubricated Hydrophobic Surfaces. Phys. Rev. Lett. 2018, 120, 244503 10.1103/PhysRevLett.120.244503. 29956993

[ref6] Armstrong S.; McHale G.; Ledesma-Aguilar R.; Wells G. G. Pinning-Free Evaporation of Sessile Droplets of Water from Solid Surfaces. Langmuir 2019, 35, 2989–2996. 10.1021/acs.langmuir.8b03849. 30702296

[ref7] Wong T. S.; Kang S. H.; Tang S. K.; Smythe E. J.; Hatton B. D.; Grinthal A.; Aizenberg J. Bioinspired self-repairing slippery surfaces with pressure-stable omniphobicity. Nature 2011, 477, 443–447. 10.1038/nature10447. 21938066

[ref8] Smith J. D.; Dhiman R.; Anand S.; Reza-Garduno E.; Cohen R. E.; McKinley G. H.; Varanasi K. K. Droplet mobility on lubricant-impregnated surfaces. Soft Matter 2013, 9, 1772–1780. 10.1039/C2SM27032C.

[ref9] Blake T. D. The physics of moving wetting lines. J. Colloid Interface Sci. 2006, 299, 7 10.1016/j.jcis.2006.03.051. 16631781

[ref10] de Gennes P.-G.; Brochard-Wyart F.; Quéré D. Capillarity and Wetting Phenomena, 1st ed.; Springer: New York, 2004.

[ref11] Bonn D.; Eggers J.; Indekeu J.; Meunier J.; et al. Wetting and spreading. Rev. Mod. Phys. 2009, 81, 739–805. 10.1103/RevModPhys.81.739.

[ref12] Voinov O. V. Hydrodynamics of wetting,. Fluid Dynamics 1977, 11, 714–721. 10.1007/BF01012963.

[ref13] Cox R. G. The dynamics of the spreading of liquids on a solid surface. Part 2. Surfactants. J. Fluid Mech. 1986, 168, 195–220. 10.1017/S0022112086000344.

[ref14] Blake T. D.; Haynes J. M. Kinetics of liquid liquid displacement. J. Colloid Interface Sci. 1969, 30, 421–423. 10.1016/0021-9797(69)90411-1.

[ref15] de Ruijter M. J.; De Coninck J.; Blake T. D.; Clarke A.; Rankin A. Contact angle relaxation during the spreading of partially wetting drops. Langmuir 1997, 13, 7293–7298. 10.1021/la970825v.

[ref16] Launay G. PyDSA Droplet Shape Analysis in Python, 2018.

[ref17] Vuckovac M.; Latikka M.; Liu K.; Huhtamäki T.; Ras R. H. A. Uncertainties in contact angle goniometry. Soft Matter 2019, 15, 7089–7096. 10.1039/C9SM01221D. 31453607

[ref18] Liu K.; Vuckovac M.; Latikka M.; Huhtamäki T.; Ras R. H. Improving Surface-Wetting Characterization. Science 2019, 3, 1147–1148. 10.1126/science.aav5388. 30872505

[ref19] Lam C.; Wu R.; Li D.; Hair M.; Neumann A. Study of the advancing and receding contact angles: liquid sorption as a cause of contact angle hysteresis. Adv. Colloid Interface Sci. 2002, 96, 169–191. 10.1016/S0001-8686(01)00080-X. 11911113

[ref20] McHale G.; Shirtcliffe N. J.; Newton M. I. Contact-angle hysteresis on super-hydrophobic surfaces,. Langmuir 2004, 20, 10146–10149. 10.1021/la0486584. 15518506

[ref21] Gao L.; McCarthy T. J. Contact Angle Hysteresis Explained. Langmuir 2006, 22, 6234–6237. 10.1021/la060254j. 16800680

[ref22] Eral H. B.; ’t Mannetje D. J. C. M.; Oh J. M. Contact angle hysteresis: a review of fundamentals and applications. Colloid Polym. Sci. 2013, 291, 247–260. 10.1007/s00396-012-2796-6.

[ref23] Shirtcliffe N. J.; McHale G.; Atherton S.; Newton M. I. An introduction to superhydrophobicity. Adv. Colloid Interface Sci. 2010, 161, 124–138. 10.1016/j.cis.2009.11.001. 19944399

[ref24] Cazabat A. M.; Guéna G. Evaporation of macroscopic sessile droplets. Soft Matter 2010, 6, 2591–2612. 10.1039/b924477h.

[ref25] Teisala H.; Baumli P.; Weber S. A.; Vollmer D.; Butt H. J. Grafting Silicone at Room Temperature-a Transparent, Scratch-resistant Nonstick Molecular Coating. Langmuir: ACS J. Surfaces and Colloids 2020, 36, 4416–4431. 10.1021/acs.langmuir.9b03223. PMC7191751 32239949

